# ALH Inhibition as a Molecular Initiating Event in the Adverse Outcome Pathway of Benomyl Toxicity in *Caenorhabditis elegans*: Relevance for Parkinsonism

**DOI:** 10.3390/ijms26189163

**Published:** 2025-09-19

**Authors:** Lucía Eugenia Fernandez-Hubeid, Romina Deza-Ponzio, Paula Alejandra Albrecht, Verónica Leonor Romero, Candelaria Gonzales-Moreno, Melisa Rut Ferreyra, Yanina Soledad Moran, Miriam Beatriz Virgolini

**Affiliations:** 1 Departamento de Farmacología Otto Orsingher, Facultad de Ciencias Químicas, Universidad Nacional de Córdoba, Córdoba X5000HUA, Argentina; luciahubeid@unc.edu.ar (L.E.F.-H.); rdezaponzio@unc.edu.ar (R.D.-P.); paula.albrecht@unc.edu.ar (P.A.A.); veronica.romero@unc.edu.ar (V.L.R.); candelaria.gonzales@unc.edu.ar (C.G.-M.); melisa.ferreyra@unc.edu.ar (M.R.F.); yanina.moran@unc.edu.ar (Y.S.M.); 2 Instituto de Farmacología Experimental de Córdoba-Consejo Nacional de Investigaciones Científicas y Técnicas (IFEC-CONICET), Córdoba X5000HUA, Argentina

**Keywords:** benomyl, *Caenorhabditis elegans*, ALDH, parkinsonism, dopamine

## Abstract

Dithiocarbamate fungicides, including benomyl (methyl 1-butylcarbamoyl-2-benzimidazolecarbamate), share a common mechanism of toxicity by inhibiting aldehyde dehydrogenases (ALDHs), enzymes essential for detoxifying reactive aldehydes. One such aldehyde, 3,4-dihydroxyphenylacetaldehyde (DOPAL), a dopamine metabolite, is implicated in the catecholaldehyde hypothesis of Parkinson’s disease. This study examines ALDH inhibition as the molecular initiating event (MIE) within an adverse outcome pathway (AOP) leading to neurotoxicity. *Caenorhabditis elegans* at the L4 stage were exposed for 24 h to 10 or 100 μM benomyl. While 10 μM had no significant effect on lethality, growth, or reproduction, 100 μM induced adverse effects, albeit with low lethality. Both doses inhibited ALH activity, an effect mitigated by Alda-1, a selective ALDH activator. Alda-1 alone increased ALH-1 protein levels but did not alter benomyl-induced protein localization and relative abundance. Benomyl exposure also elevated oxidative stress markers—superoxide dismutase, catalase, and lipid peroxidation—which Alda-1 reduced. Neurotoxicity was evidenced by dopaminergic dysfunction, including impaired basal slowing response, neuronal morphological abnormalities, and reduced locomotion upon optogenetic activation. Fluorescent reporter assays confirmed ALH-1 presence in dopaminergic neurons. These results identify ALH inhibition as the MIE in benomyl-induced neurotoxicity, linking dopaminergic degeneration and redox imbalance to the catecholaldehyde hypothesis, and providing mechanistic insights into an AOP relevant to neurodegenerative disorders.

## 1. Introduction

The widespread use of pesticides presents a paradox for humanity: on one hand, they are essential for effective pest control aimed at meeting the growing food demands of a rising global population, but on the other, their intrinsic toxicity poses significant environmental and health risks to humans and non-target species [1]. Thus, understanding pesticide toxicity mechanisms is crucial for identifying early biomarkers, enabling early diagnosis, monitoring, and mitigating their adverse effects in living organisms.

Benomyl (methyl 1-butylcarbamoyl-2-benzimidazole-carbamate), a broad-spectrum fungicide, has relatively low acute toxicity (oral LD_50_ > 10,000 mg/kg in rats) but is considered a possible carcinogen by the International Agency for Research on Cancer (IARC) [2]. Among the few studies that evaluated this pesticide, it has been reported that SHSY-5Y cells incubated with 1–6 µM benomyl for 24 h revealed dose-dependent oxidative stress, DNA damage, and apoptosis [3]. Coincidentally, it also induced death in other cell types, such as MCF-7, HTR-8/SVneo, and HeLa [4,5,6]. Therefore, due to concerns about its safety, benomyl was deregistered in the United States and the European Union [7]. However, its use remains authorized in several countries under the designation of carbendazim, its active metabolite [8,9]. In humans, benomyl undergoes bioactivation to carbendazim and butyl isocyanate, followed by metabolism into S-methyl N-butylthiocarbamate (MBT), and further oxidation by cytochrome P450 enzymes into S-methyl N-butylthiocarbamate sulfoxide (MBT-SO), a potent inhibitor of aldehyde dehydrogenase (ALDH) [10,11].

ALDHs are NAD^+^ or NADP^+^-or NADP-dependent enzymes essential for aldehyde detoxification. Nineteen human ALDH genes have been characterized, with isoforms localized in the cytosol, mitochondria, endoplasmic reticulum, and nucleus [12,13]. ALDH1A1 is highly expressed in dopaminergic neurons and detoxifies lipid peroxidation-derived aldehydes such as 4-hydroxy-2-nonenal (4-HNE) and malondialdehyde (MDA). ALDH2, located in the mitochondrial matrix, is broadly expressed and metabolizes acetaldehyde (Km < 1 μM) and 3,4-dihydroxyphenylacetaldehyde (DOPAL; Km = 4.2 μM), a dopamine-derived aldehyde [14,15]. Reactive aldehydes exert cytotoxic effects by forming adducts with proteins, enzymes, and nucleic acids, and by generating reactive oxygen species (ROS) through redox cycling, promoting lipid peroxidation and oxidative stress [16]. This redox imbalance amplifies DOPAL accumulation, further inhibiting ALDH activity and contributing to dopaminergic neuron loss [17,18,19,20,21]. Pharmacological ALDH activation, e.g., with Alda-1 (N-(1,3-benzodioxol-5-ylmethyl)-2,6-dichlorobenzamide), enhances aldehyde detoxification by stabilizing the tetrameric structure of the enzyme [22,23], increasing activity and expression to promote neuroprotection [24].

In *Caenorhabditis elegans*, ALDH enzymes (ALHs) are key in aldehyde detoxification and redox homeostasis [25,26]. WormBase (https://wormbase.org) identifies 13 *alh* genes, with *alh-1* and *alh-10* oxidizing 4-HNE [27,28], *alh-6* and *alh-10* participating in ethanol metabolism, and *alh-4* regulating fatty aldehyde levels. Notably, *alh-1* and *alh-2* are critical for neuronal detoxification; *alh-1* is broadly expressed, including in neurons, while *alh-2* is more restricted, notably in dopaminergic neurons. ALH-1A and ALH-1B share 100% sequence identity and 82–85% homology with ALH-2. All isoforms show greater similarity to human ALDH2 than to ALDH1A1 or ALDH1B1 (see Supplementary Data).

Experimental data show that inhibition of ALDH1A1 and ALDH2 by dithiocarbamates promotes toxic aldehyde accumulation and cell death [29,30]. In *Danio rerio*, benomyl selectively damages dopaminergic neurons, impairing locomotion [31]. Further studies identified ALDH2 genetic variants that increase Parkinson’s disease (PD) risk when exposed to ALDH-inhibiting pesticides [32]. Benomyl rapidly inhibits ALDH activity, causing DOPAL accumulation in vivo (mouse striatum) and in vitro (PC12 cells, fibroblasts, glia) [33,34]. Similar DOPAL accumulation and neurotoxicity occur in mouse PD models after ALDH1A1/ALDH2 knockdown [35], supporting the catecholaldehyde hypothesis [36]. While dithiocarbamates are neurotoxic in *C. elegans* [37], benomyl’s effects in this model have not been previously explored.

*C. elegans* has a well-characterized dopaminergic system [38,39,40] composed of eight neurons (2 ADE, 4 CEP, 2 PDE), ideal for studying the dopamine system [41,42]. Behavioral assays such as the basal slowing response (BSR), where worms reduce movement upon sensing food, are used to assess dopamine signaling [43,44]. Optogenetic activation via Channelrhodopsin-2 in Pdat-1::ChR2 transgenic worms allows evaluation of dopaminergic function independently of food cues [45,46].

Building upon this evidence, we propose that benomyl-induced inhibition of ALH enzymes in *C. elegans* leads to the accumulation of toxic aldehydes and redox imbalance. These effects are expected to be attenuated by ALDH activators such as Alda-1. Together with dopaminergic dysfunction, this mechanism may reproduce features of neurodegeneration, positioning *C. elegans* as a useful model to investigate the catecholaldehyde hypothesis and underlying pathways relevant to Parkinsonian disorders.

## 2. Results

### 2.1. Physiological and Morphological Parameters

**Figure 1:**
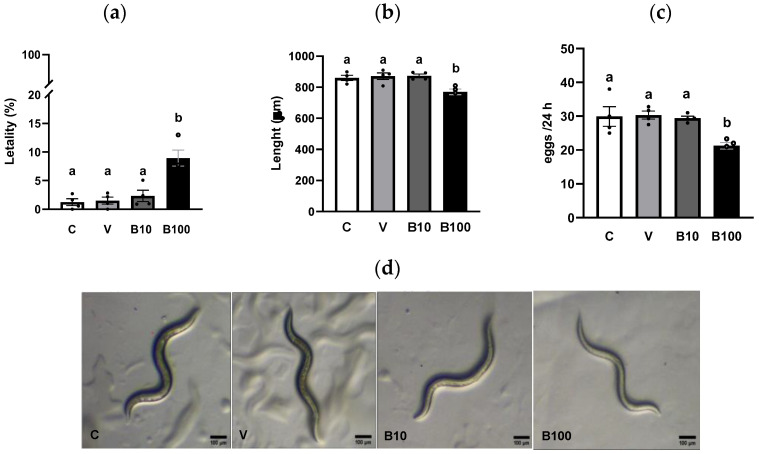
(**a**) Lethality. (**b**) Size. (**c**) Number of eggs. (**d**) Representative photography (10×) of *C. elegans* from each group. Different letters indicate differences between groups at *p* < 0.001 (panel (**a**)); *p* < 0.001: C and V vs. B100 and *p* < 0.01 B10 vs. B100 (panel (**b**)). *p* < 0.05: C, V and B10 vs. B100 (panel (**c**)). C = control; V = vehicle; B10 = 10 μM benomyl; B100 = 100 μM benomyl.

Figure 1a shows the percentage of dead animals at the end of treatment with benomyl. A small lethality index was observed in the control and vehicle groups, demonstrating the absence of toxicity under basal conditions, which increased in response to the higher concentration of benomyl. A one-way ANOVA revealed a significant difference between groups (*p* < 0.001), due to the higher mortality of animals exposed to 100 μM benomyl compared to the other groups. Based on these results, and considering that lethality in the group exposed to the highest concentration of benomyl, while significantly greater than at 10 μM, remained below 10%, both fungicide concentrations were selected for subsequent experiments designed to assess toxicity across multiple parameters under sublethal exposure conditions. The size of the animals at the end of exposure to both doses of the fungicide is graphed in Figure 1b. A one-way ANOVA showed a significant effect between groups (*p* < 0.001) as a result of the slower growth of animals exposed for 24 h to the 100 μM benomyl concentration. Regarding egg laying, a one-way ANOVA revealed a significant interaction (*p* < 0.01), due to the lower number of eggs recorded at the end of exposure to the highest dose of the fungicide (Figure 1c). These results may be attributed to benomyl’s interference with normal growth and development in the animals.

### 2.2. Aldehyde Dehydrogenase Functionality and Redox Indicators

#### 2.2.1. Aldehyde Dehydrogenase Activity

After exposure to 10 µM or 100 µM benomyl alone or concomitantly with 20 µM Alda-1 activator, the collected and washed N2 nematode pellets were stored at −80 °C.

**Figure 2:**
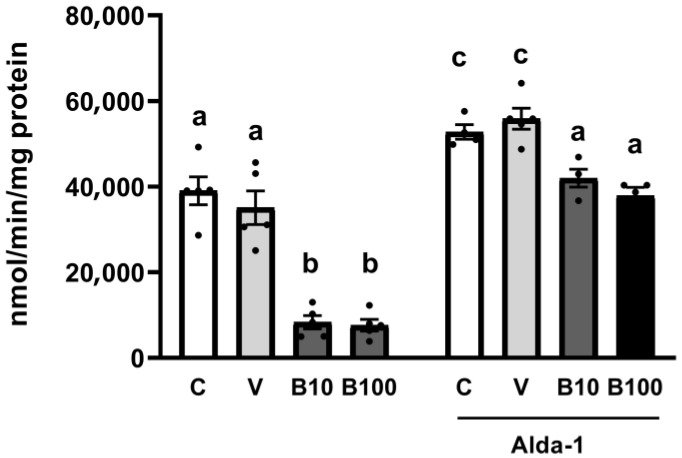
ALH activity of the groups exposed to benomyl (**left**) and of the groups exposed to benomyl and treated with the Alda-1 activator (**right**). Different letters indicate differences between groups at *p* < 0.05 (a vs. c for the C and V −/+ Alda-1 groups) and *p* < 0.01 (b vs. a for the B10 and B100 −/+ Alda-1 groups). C = control; V = vehicle; B10 = 10 μM benomyl; B100 = 100 μM benomyl.

The results presented in Figure 2 reveal that benomyl, at the two concentrations tested, drastically inhibited ALH activity compared to the control and vehicle groups. The absence of a dose-related effect on this output may be associated with enzyme saturation or compensatory mechanisms, evidencing the activity inhibition as a plateau effect. Interestingly, co-exposure with Alda-1 significantly increases the enzyme’s activity in all groups, with the most evident effect evidenced in the groups of animals exposed to both concentrations of the fungicide.

#### 2.2.2. Aldehyde Dehydrogenase Localization and Relative Abundance

**Figure 3:**
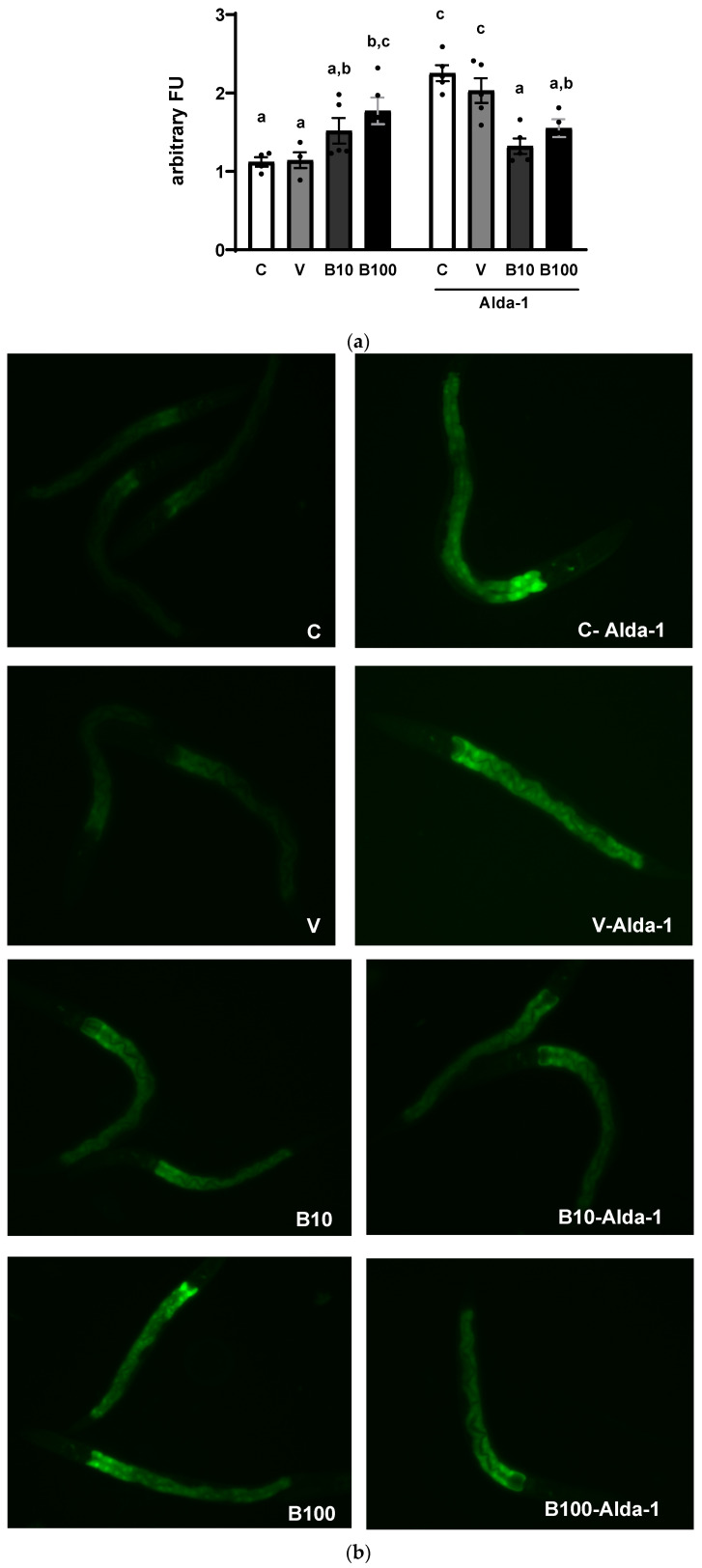
(**a**) ALH-1 protein localization and relative abundance in the BC11202 strain from groups exposed to benomyl alone (**left**) or benomyl plus Alda-1 (**right**). (**b**) Representative photographs (20×) of *C. elegans* in all experimental groups. Different letters indicate differences between groups; *p* < 0.05. C = control; V = vehicle; B10 = 10 μM benomyl; B100 = 100 μM benomyl.

Figure 3 shows the semi-quantification of ALH-1 protein expression (panel a) and representative photographs (panel b). The two-way ANOVA revealed a significant interaction: F (3, 29) = 14.37; *p* < 0.05 between the variable groups and treatment. The highest dose of benomyl induced, in conditions without Aldehyde-1, an increase in ALH-1 relative protein levels, contrary to what we reported for the activity of this enzyme in Figure 2, probably due to a compensatory effect of increased protein synthesis in response to the inhibition of enzymatic activity induced by the fungicide. Interestingly, co-exposure to Alda-1 increased ALH-1 localization and relative abundance selectively in the control and DMSO groups (but not in the benomyl-exposed groups) as demonstrated by the significant differences emerging from the statistical contrast between groups and visualized in the pictures obtained by fluorescence microscopy.

#### 2.2.3. Catalase and Superoxide Dismutase Activity

**Figure 4:**
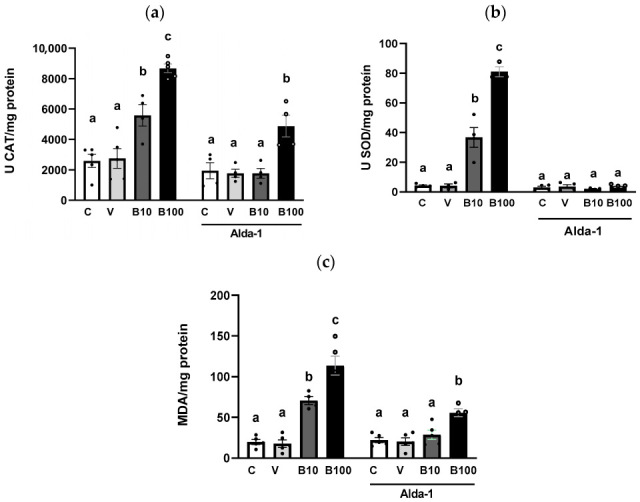
(**a**) Catalase and (**b**) superoxide dismutase activities. (**c**) Lipid peroxidation index expressed as MDA/mg protein. Different letters indicate differences between groups at *p* < 0.01 (panel (**a**)), *p* < 0.001 (panel (**b**)), and *p* < 0.001 (panel (**c**)). C = control; V = vehicle; B10 = 10 μM benomyl; B100 = 100 μM benomyl. U = enzymatic units.

The results for catalase activity expressed as enzymatic units per mg of protein are shown in Figure 4a. The two-way ANOVA showed an interaction between all parameters that reached statistically significant differences: (F (3, 27) = 5.66; *p* < 0.01), demonstrating greater enzyme activity in the groups exposed to benomyl, which was reduced in response to Alda-1, an effect that was absent in the control and vehicle groups.

Regarding SOD determination expressed as enzymatic units per mg of protein (Figure 4b), the two-way ANOVA showed a significant interaction between groups and treatment (F (3,22) = 78.86; *p* < 0.001), accompanied by significant differences for each factor (*p* < 0.001). Thus, exposure to benomyl significantly increased enzyme activity relative to controls, an effect that disappeared in the presence of Alda-1.

#### 2.2.4. Lipid Peroxidation

The results plotted in Figure 4c show an increase in MDA generation as an index of lipid peroxidation in response to exposure to the two concentrations of benomyl. A two-way ANOVA was performed, revealing significant interactions (F (3,30) = 13.05; *p* < 0.001), resulting from both the increase in lipid peroxidation induced by the two concentrations of benomyl and the reduction in these levels in response to Alda-1 treatment.

### 2.3. DA Functionality Assessment

#### 2.3.1. Basal Slowing Response

**Figure 5:**
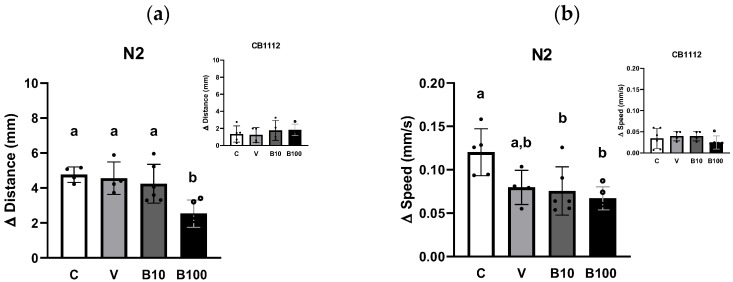
Locomotor activity expressed as the difference in distance traveled (**a**) or the difference in speed (**b**) recorded during 1 min in the absence or presence of food in animals of N2 strain. The insert represents the same data for the CB1112 strain. Different letters indicate differences between groups; *p* < 0.05. C = control; V = vehicle; B10 = 10 μM benomyl; B100 = 100 μM benomyl.

Figure 5a shows the difference in walking distance between the N2 (a) and CB1112 strains (insert c) between the fed and fed conditions, demonstrating the absence of differences related to the presence of food between the two strains (*p* = 0.74). A one-way ANOVA revealed significant differences in walking distance at *p* = 0.01 for each strain.

Figure 5b shows the difference in walking speed considering the feed/food absence conditions for N2 (a) and CB1112 strains (insert c). Since the two-way ANOVA failed to establish a significant interaction between strains and experimental groups, the results were compared using one-way ANOVA, analyzing each strain separately. Wild-type animals showed a statistically significant effect: *p* < 0.05, due to the difference between the benomyl-exposed and control groups.

As expected, the results demonstrated a difference compared to the control group in both conditions (with and without food) in locomotor activity assessed as distance traveled and speed. Locomotion was greater in the no-food condition, as evidenced by the animals’ ability to discriminate a surface with food and reduce their locomotion by approximately 60%. Finally, as expected, this difference in locomotion was less evident in the CB1112 strain tested as a control, since it presents a mutation for the TH enzyme with DA levels between 30 and 40% compared to the wild-type strain [44].

#### 2.3.2. Dopaminergic Neuron Morphology

**Figure 6:**
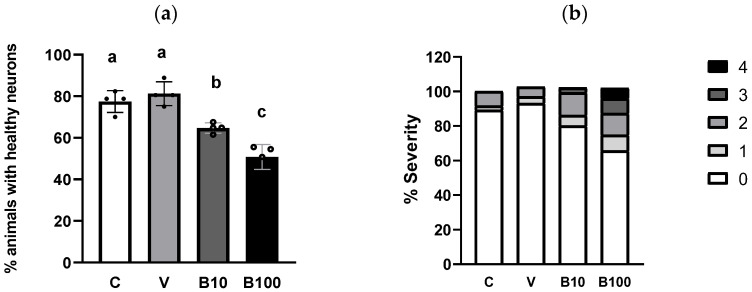

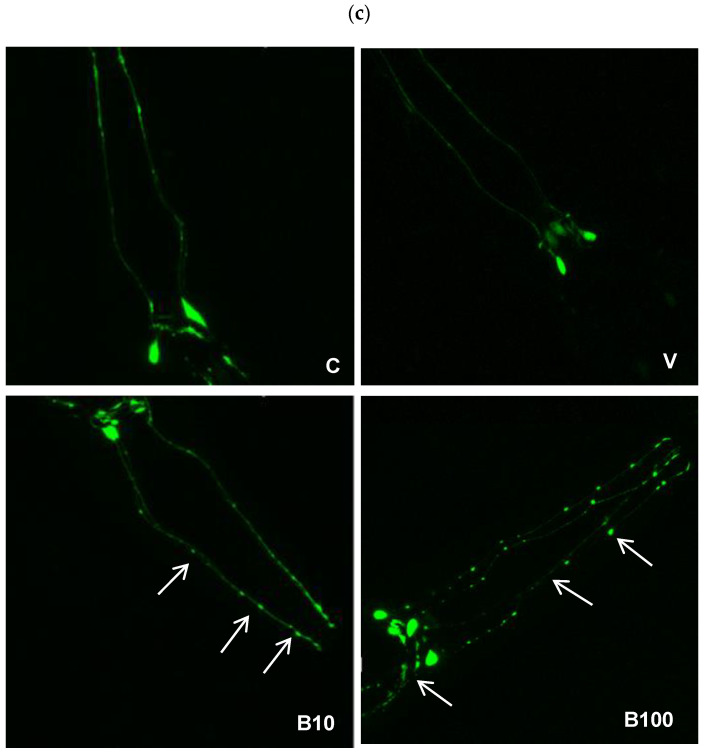
(**a**) Percentage of animals with healthy neurons evidenced in strain BZ555 in response to exposure to the two concentrations of benomyl. (**b**) Severity of dendritic damage expressed as the number of worms that presented a specific type of alteration according to the scale described in Bijwadia et al., 2021 [48]. (**c**) Representative photographs of the morphological changes in dopaminergic cephalic neurons. (**a**,**b**): Different letters indicate differences between groups at *p* < 0.05. Neuromorphological alterations are indicated by the white arrows in each panel. C = control; V = vehicle; B10 = 10 μM benomyl; B100 = 100 μM benomyl.

Figure 6 shows the percentage of dendrites in the four cephalic dopaminergic neurons from the BZ555 strain. Panel 6a depicts the benomyl-induced decrease in animals showing normal neuron morphology, with signs of neuronal damage evidenced as discontinuities and bubbles in the CEP neurons’ projections. This effect was dose-dependent in the groups exposed to benomyl, with a significant interaction at *p* < 0.01 obtained in the one-way ANOVA. Regarding the severity of the morphological alterations, panel 6b shows that approximately 20% of the control animals show mild neuronal morphological modifications according to the scale used. This finding may be linked to the presence of vesicles in dopaminergic CEP neurons associated with the normal aging process observed in adult nematodes [47], in this case, the L4 larval stage. Regarding the groups treated with 10 µM benomyl, approximately 65–70% of animals had healthy neurons, while in those exposed to 100 µM benomyl, this number decreased to approximately 50–55%. Thus, the highest concentration of the fungicide induced signs of morphological alteration of greater severity than 10 µM benomyl, evidenced as a greater number of interruptions, bubbles, and protrusions, considerably affecting the continuity of the evaluated dendrite (pictured in panel 6c). These results reveal the impact of this fungicide on neuronal morphology, which could have direct implications for the functionality of the dopaminergic system and eventually related aminergic systems.

#### 2.3.3. Optogenetic Stimulation

**Figure 7:**
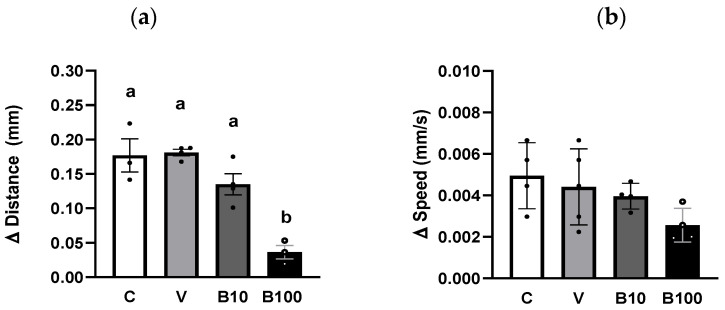
Locomotor activity expressed as the difference between the conditions without and with light in the distance traveled (**a**) and speed (**b**) recorded during 1 min in animals of the ZX909 strain. Different letters indicate differences between groups at *p* < 0.001. C = control; V = vehicle; B10 = 10 μM benomyl; B100 = 100 μM benomyl.

Figure 7 shows the difference between the light-free and light-enhanced conditions in the distance traveled (a) and speed (b) of animals of the ZX909 strain, which expresses the ChR2 protein in dopaminergic neurons under the DAT-1 transporter promoter. Consistent with recent reports [46], this assay confirms that illuminating dopaminergic neurons triggers their activation in response to the light stimulus, in turn, decreasing free locomotion. Regarding the groups exposed to the fungicide, a reduction in the locomotor response was observed in nematodes exposed to 100 µM benomyl, although not in the group exposed to the lower concentration. A one-way ANOVA was performed to test the difference in distance traveled, which showed a significant interaction effect between factors at *p* < 0.001 due to the lack of difference in this parameter between the conditions with and without light in the group exposed to 100 μM benomyl. However, no interaction between factors emerged for the speed parameter (*p* = 0.13) despite a similar trend being observed in the group exposed to the highest benomyl concentration.

### 2.4. Expression of the alh-1 Gene in Dopaminergic Neurons

**Figure 8:**
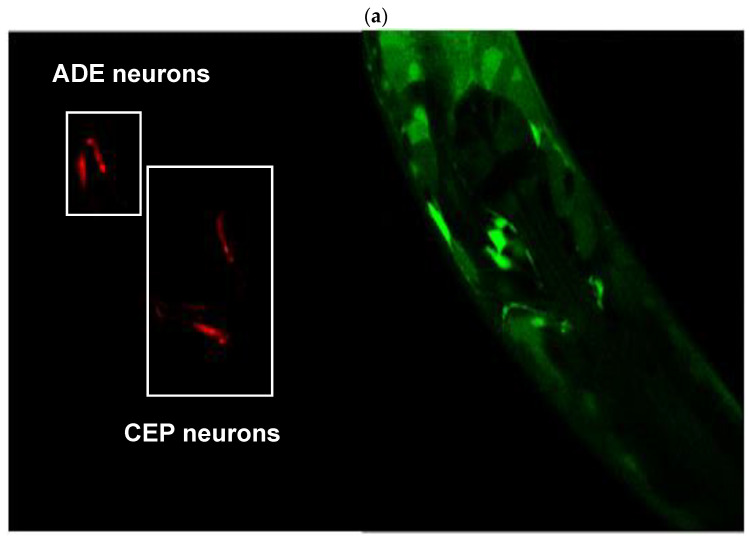

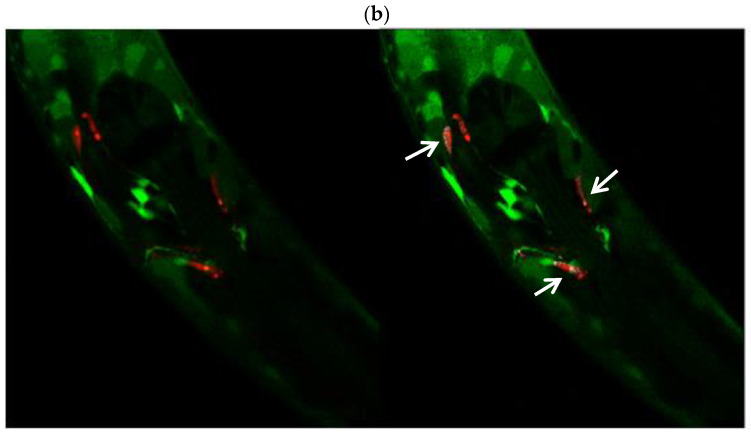
(**a**) Dopaminergic neurons labeled with mCherry (**left**) and expression of the GFP-tagged *alh-1* gene product (**right**). (**b**) Co-localization of labeling. White stippling denoted by white arrows indicates co-labeling.

Images in Figure 8 were captured by confocal microscopy of the cross between animals. The red channel (mCherry) shows the body of the dopaminergic ADE and CEP neurons (panel A, left), while the green channel (GFP) denotes the protein localization and relative abundance of the ALH-1 protein (Panel A, right). Panel B shows the co-localization of both channels (left) and the same photograph using a tool in Image J version 1.54c, which denotes the co-localization areas of both colors with white dots (right, white arrows). The photographs demonstrate the presence of the ALH-1 enzyme, a product of the *alh-1* gene, in dopaminergic neurons. Although this result does not provide quantitative values, it has implications for DOPAL metabolism due to the presence of the ADH-1 enzyme in these neurons, which has a high affinity for its substrate (Km = 4.2 μM). In this regard, although the photographs only show the head, where the ADE and CEP neurons are located, ADH-1 is an enzyme present throughout the worm’s body [49].

## 3. Discussion

In this novel study, we demonstrate for the first time the toxic effects of the fungicide benomyl in the invertebrate model *C. elegans*. We show that benomyl at 100 µM, the highest tested concentration, but not at 10 µM, significantly increases lethality, retards growth, and reduces fertility, indicating a threshold effect between both doses for these toxicity endpoints. Although these parameters do not directly assess molecular mechanisms, they are critical for determining appropriate dosing for subsequent experiments. We propose that the inhibition of the ALDH activity (Figure 2) represents the MIE in the adverse outcome pathway leading to neurotoxicity characterized by a Parkinsonian phenotype. This is a key finding because, as noted in the Introduction, benomyl must undergo bioactivation to produce MBT-SO, the thiocarbamate derivative that inhibits ALDH [11,33]. Accordingly, the present exposure protocol demonstrates a marked reduction in ALDH activity in *C. elegans*, supporting the face validity of this model organism. Importantly, as reported by Kara et al., (2020) [3], the current daily margin of exposure (MOE) for potential daily dietary exposure to benomyl in humans ranges from 342 to 1300 μg/mL (equivalent to 1.2 to 4.5 mM). This dose, although low, induces the ALDH inhibition in our study, which aligns with the established metabolic pathway reported in mammals, reinforcing its translational relevance and highlighting the risks of this fungicide for human health even at low concentrations. Moreover, the biological relevance of this inhibition is underscored by the observed compensatory upregulation of the ALH protein (Figure 3) and its mRNA (Figure S3) at the highest benomyl exposure concentration. Therefore, despite differences between *C. elegans* ALH-1/ALH-2 and mammalian ALDHs, including sequence divergence and tissue-specific expression, the interspecies homology among the different isoforms is valid, although it warrants further investigation to enhance the translational relevance of invertebrate models in toxic aldehyde-related pathologies. The aforementioned scenario was associated with elevated MDA levels, indicative of increased lipid peroxidation, alongside disrupted redox homeostasis, as evidenced by the upregulation of antioxidant enzyme activities [49,50,51,52], further corroborating the adverse effects of toxic aldehyde accumulation. In addition, the predictive validity of the *C. elegans* experimental model was demonstrated by the partial attenuation of benomyl-induced toxic effects following treatment with Alda-1 (Figure 4), facilitating ALDH2 catalytic efficiency and NAD^+^ affinity [23]. Importantly, Alda-1’s protective effect was shown to be dependent on aldehyde levels, as some studies suggest efficacy only under high aldehyde stress conditions (>5 mM) with potential opposite effects at lower levels [53]. In this context, our findings suggest an elevated aldehyde burden, highlighting the therapeutic potential of Alda-1 counteracting ALDH inhibition and promoting aldehyde detoxification.

Regarding the catecholaldehyde theory, although DOPAL levels were not measured, ALH-1 localization in dopaminergic neurons (Figure 8) and the reduction in ALH activity suggest intracellular DOPAL accumulation in these cells. Furthermore, the behavioral changes, particularly the correlate well with dopaminergic neuronal damage as reported by Clark et al., (2023) [54], whereas spontaneous locomotion and cholinergic neuron integrity remained unaffected (Figures S1 and S2), supporting the selective nature of benomyl’s neurotoxicity to dopamine neurotransmission. The use of optogenetics to activate only dopaminergic neurons further validated the functional impact of the fungicide on these cells [55]. These observations are coincidental with multiple reports demonstrating selective dopaminergic neurodegeneration, mitochondrial dysfunction, and oxidative stress associated with dithiocarbamate exposure [56,57,58,59,60]. Moreover, similarities between our findings and those from Caito et al., 2013 [37] suggest a shared neurotoxic mechanism involving ALDH inhibition and dopaminergic damage due to thiocarbamate exposures in the *C. elegans* model. In addition, epidemiological studies have associated benomyl exposure with increased risk of PD, especially in occupational and residential settings [32]. This vulnerability has been mechanistically linked to ALDH inhibition and consequent DOPAL buildup, with direct implications for the catecholaldehyde hypothesis of PD [16,31,33,61,62,63,64,65]. Finally, our findings contribute to differentiating cytotoxic pathways mediated by catecholamine autoxidation from those driven by enzymatic DOPAL formation, thereby providing support for the catecholadehyde hypothesis. Thus, if cytoplasmic catecholamines were primarily toxic due to autoxidation, ALDH inhibition would be irrelevant; however, our results are consistent with the enzymatic oxidation model, which implicates DOPAL accumulation. This is supported by evidence derived from mice with reduced ALDH1A1 and ALDH2 activity, which exhibit age-related PD-like phenotypes leading to DOPAL accumulation and α-synuclein oligomerization [66], reinforcing the etiology of the catecholaldehyde hypothesis.

## 4. Materials and Methods

### 4.1. Animals and Strains

Nematodes were cultured and maintained in NGM medium in Petri dishes seeded with *E. coli* OP50 as a food source in a temperature-controlled incubator at 20 °C. All studies were performed in duplicate on animals at the L4 larval stage obtained from the synchronization process [67]. The number of replications is denoted by dots in each Figure. In all experiments (except where otherwise noted), the strain used was N2. Other strains tested were: CB1112 (cat-2(e1112) II); LX929 (vsIs48 [unc-17::GFP]); BZ555 (egIs1 [dat-1p::GFP]); BC11202 (sIs10158 [rCesF54D8.3::GFP) y OH7193 (otIs181 [dat-1::mCherry + ttx-3::mCherry]) from the Caenorhabditis Genetic Center (CGC, MN, EE.UU.) and the ZX909 strain (Lite1(ce314);zxIs20 [pdat-1::ChR2(H134R)::mCherry;pmyo-2::mCherry generously donated by Dr. A. Gottschalk.

### 4.2. Exposure Protocol

A stock solution was prepared with a benomyl concentration of 10 mM (CAS 17804-35-2PESTANAL®, analytical standard, Sigma Aldrich, Argentina) using 1% DMSO (CAS 67-68-5, Sigma Aldrich, Argentina) as the vehicle; this solution was separated into aliquots and stored at −20 °C until use. Based on the thiocarbamate derivatives reported in Caito et al., 2013 [37], dilutions were performed immediately before the experiments, achieving final concentrations of 10 µM and 100 µM in M9 buffer. Exposure to benomyl alone or concomitantly with the activator Alda-1 (in appropriate groups) was performed with constant shaking for 24 h in a population of synchronized L4 stage animals in 24-well plates containing M9 buffer.

### 4.3. Physiological and Morphological Parameters

After exposure, the animals were immediately washed with M9 solution twice and transferred to plates with NGM medium without food to be observed using a magnifying glass to record the following parameters:

#### 4.3.1. Lethality

The number of live and dead animals was determined following the protocol of Helmcke et al., 2010 [68], considering the presence or absence of animal response to a gentle touch with the platinum loop.

#### 4.3.2. Growth

Measurement of worm length, indicative of growth in response to benomyl exposure, was recorded using photographs of 10–15 individuals per experimental group with a Moticam loupe (Motic SMZ-171, Tecno Edu, Argentina) and analyzed with Image J version 1.54c, software (NIH, Washington DC, USA), according to the protocols reported by Meyer et al., 2010 [69].

#### 4.3.3. Reproduction

Five to 10 individuals from each group were transferred to 3 cm NGM plates with a thin layer of food. Twenty-four hours later, according to Rui et al., (2013) and Moya et al., (2022) [70,71], litter size was recorded by manually counting the number of eggs deposited on a gridded template under a Moticam stereoscope (Motic SMZ-171, Tecno Edu, Argentina).

### 4.4. Aldehyde Dehydrogenase Functionality and Redox Indicators

The following experiments sought to describe ALH activity and relative levels, superoxide dismutase (SOD) and catalase (CAT) activities as main enzymatic antioxidant defense, as well as lipid peroxidation index in response to benomyl exposure alone or in combination with the ALH activator Alda-1.

#### 4.4.1. ALH Activity

Upon completion of exposure to benomyl 10 μM or 100 μM alone or in concomitance with Alda-1 20 μM activator, animals were immediately washed with M9 solution twice and sonicated in phosphate buffer at pH = 7, following procedures described in Ferreyra et al., 2024 [72]. The selection of the 20 μM concentration was based on previous results in SHSY-5Y cells in which a marked increase in ALDH activity was reported [73]. Samples were then centrifuged at 10,000× *g* for 5 min, and the supernatant was collected and stored at −80 °C until biochemical determinations were performed. Protein concentrations in the samples were determined using the Bradford assay (1976) [74] with values calculated based on a standard curve generated from serial dilutions of a known standard of bovine serum albumin.

Once thawed, the pellet of worms exposed to benomyl and/or Alda-1 was lysed with the addition of 400 µL PBS buffer, sonicated, and centrifuged for 10 min at 800 g at 4 °C. The supernatant was collected and centrifuged at 12,000× *g* for 10 min at 4 °C, then discarded, and the resulting pellet resuspended in 500 µL of 0.25 M sucrose and 1% Triton solution. The final homogenate was frozen at −80 °C for 30 min and then centrifuged again at 10,000× *g* for 10 min before determination of ALH activity as reported in Deza-Ponzio et al., 2023 [73]. For this aim, 500 µL of the reaction mixture (2 mL sodium pyrophosphate 50 mM, pH 8.8; 0.01 mL rotenone 2 µM; 1 mL NAD 1 mM; 1 mL MgCl2 1 mM; 0.1 mL 4-methylpyrazole 0.2 mM; 5 mL sucrose 0.25 M s.c.) was incubated with 300 µL of the worm homogenate for 10 min. Next, 100 µL of acetaldehyde was added, and the absorbance at 340 nm, determined by the appearance of NADH, was read for 10 min every 1 min for a total of 10 consecutive measurements. ALH activity was expressed as nmol NADH/mg protein/min.

#### 4.4.2. Aldehyde Dehydrogenase Localization and Relative Abundance

Animals of strain BC11202 subjected to the exposure protocol were collected, washed, and mounted on 2% agarose pads using 5 mM levamisole (CAS 16595-80-5, Sigma Aldrich, Argentina) as an anesthetic. Images of 10–15 individuals per experimental group were taken at 20× magnification under a microscope (Leica DMI 8, BIO.-OPTIC). The fluorescence intensity of the images obtained was analyzed with Image J version 1.54c software (NIH, Washington, DC, USA), the result being expressed in arbitrary fluorescence units.

#### 4.4.3. Catalase

After sample preparation, as mentioned before, enzyme activity was determined according to the method of Aebi (1984) [75] following the decomposition of H_2_0_2_ evidenced by the decrease in absorbance at a wavelength of 240 nm. Briefly, 1.25 mL of phosphate buffer (50 mM; pH = 7), 50 µL of the worm supernatant, and 100 µL of H_2_0_2_ 100 µM were added. The absorbance was measured immediately after the addition of H_2_0_2_ and 5 min after the start of the reaction. The results were expressed as enzymatic units per mg of protein of CAT/mg protein/min, considering the molar extinction coefficient of H_2_O_2_ e_240_ = 0.0394 mmol^−1^.

#### 4.4.4. Superoxide Dismutase

Following sample preparation as mentioned before, enzyme activity was determined based on its ability to inhibit the photochemical reduction in tetrazolium blue (NBT) at 560 nm according to the method of Beauchamp & Fridovich (1971) [76]. Briefly, the reaction mixture was obtained by adding 300 µL of 13 mM methionine, 100 µL of 75 µM NBT, 300 µL of 0.1 nM EDTA, 300 µL of 2 µM rivoflavin, and 100 µL of the worm supernatant. This reagent mixture was incubated for 1 h in the dark, and the absorbance was determined at 560 nm. One unit of SOD was defined as the amount of enzyme required to produce a 50% inhibition of NBT reduction under the specified conditions.

#### 4.4.5. Lipid Peroxidation

Peroxidized lipids determination was based on the formation of the MDA adduct (metabolite product of lipid peroxidation) with thiobarbituric acid (TBARs), which can be quantified spectrophotometrically [77]. For this purpose, 100 µL of the worm supernatant, 100 µL of 8% SDS, 750 µL of 20% acetic acid, pH 3.5, 750 µL of 0.8% TBA reagent, and 2 mL of distilled H_2_O were added to the reaction mixture and incubated for 1 h at 95 °C. Next, the reaction tube was centrifuged for 10 min at 3000 rpm, and the absorbance of the upper layer was measured at 532 nm. For the calibration curve, 1,1,3,3-tetramethoxypropane was used as a standard, while the results were expressed as nmol MDA/mg protein.

### 4.5. DA Functionality Assessment

#### 4.5.1. Basal Slowing Response

The experimental procedure was set up according to Albrecht et al. (2022) [78], adapted from Chakraborty et al., 2015 [79]. At the end of benomyl exposure, 10–15 N2 animals were transferred to plates with NGM medium. After 5 min of habituation, speed and distance traveled for 1 min were measured using WMicrotacker Smart device software (Phylumtech SA, Argentina). The results were expressed as the difference in distance traveled or speed in the absence and presence of food for both the N2 animals and the CB1112 strain, which was considered a positive control for this behavior, provided the low dopamine content resulting from the TH null mutation.

#### 4.5.2. Dopaminergic Neuron Morphology

At the end of benomyl exposure, animals of the BZ555 strain were collected, washed, transferred, and maintained for 24 h on NGM plates with food. The next day, immediately before microscopic visualization, 30–50 animals were mounted on 2% agarose pads using 5 mM levamisole (CAS 16595-80-5, Sigma Aldrich, Argentina) as an anesthetic. After 5 min, the dopaminergic neurons were observed under a confocal microscope at 60× (Olympus FV1200, Argentina) for morphological assessment of the 4 CEP neurons according to the protocol described by Bijwadia et al., 2021 [48], and Palikaras et al., 2022 [80], using the following scale for classification according to the observed alterations:

0—no visible damage, “perfect” neurons.

1—irregular (kinks, curves, etc.).

2—less than 5 spots, or bubbles.

3—5–10 spots or bubbles.

4—more than 10 bumps and/or breakage, removing more than 25% of the total dendrite.

5—breakage, 25–75% of the dendrite removed.

6—breakage, more than 75% of the dendrite removed.

#### 4.5.3. Optogenetic Stimulation

The protocol was adapted from Pokala et al., 2018, Oranth et al., 2018, and Koopman et al., 2021 [46,81,82], with slight modifications. Exposed, collected, and washed animals of strain ZX909 were transferred to plates with food in the absence or presence of 5 µL All-trans-retinal (ATR; CAS 116-31-4), Sigma Aldrich, Argentina) 100 mM added to 1 mL of bacterial culture for 24 h. Transgenic worms were used to assess light activation of dopaminergic neurons, while those cultured in the absence of ATR were used as a negative control. The plates were kept in the dark due to the liability of ATR to light. After 24 h, between 5 and 10 individuals from each experimental group were transferred to the center of a plate with NGM medium without food, where they were allowed to habituate for 5 min and then filmed free locomotion for 1 min, of which the first 30 s were in the absence of the light stimulus. The plate was then directly illuminated for the next 30 s with a 450 nm wavelength LED lamp placed at a 45-degree angle to illuminate the center of the plate and at a distance of 5 cm for all tests. The distance traveled and velocity under conditions of absence or presence of light were calculated from the protocol published in Albrecht et al., 2022, and Fernandez-Hubeid et al., 2024 [83,84].

### 4.6. Expression of the alh-1 Gene in Dopaminergic Neurons

To demonstrate the expression of the *alh-1* gene (and ALH presence) in dopaminergic neurons of worms not exposed to benomyl, a cross of hermaphrodite worms of the BC11202 reporter strain was performed, which presents GFP for visualization of the alh-1 gene product, and male worms of strain the OH7193 strain expressing a red reporter protein (mCherry) under the dat-1 promoter, allowing visualization of dopaminergic neurons in red.

Between 5 and 10 hermaphrodites of the BC11202 strain were plated on 10 plates with NGM medium and *E. coli* OP50, with 2 males of strain OH7193. At the end of the initial cross, L4 stage hermaphrodite nematodes showing green and red fluorescence in their phenotype were traced on the F1 plate. Ten individuals with these characteristics were selected and transferred to new plates with food, with one worm per plate for self-fertilization. Finally, after 48 h, the animals of the new population (F2) were mounted on 2% agarose pads using 5 mM levamisole (CAS 16595-80-5, Sigma Aldrich, Argentina) as an anesthetic to evaluate co-localization of the labeling of both fluorescent proteins. Using a confocal microscope (Olympus FV1200, Argentina), dopaminergic neurons were localized at 60× using the mCherry marker as a guide. After channel opening to visualize GFP, photographs were taken in both channels and the Z-plane to co-localize the labeling.

### 4.7. Statistical Analysis

Data analysis and graphs presented were performed in GraphPad Prism 8 software (GraphPad Software, San Diego, CA, USA). In all cases, the results were analyzed by one-way (group) or two-way ANOVAs (group vs. x treatment − presence/absence of Alda-1) followed by Tukey’s test as post hoc in case of significant interaction between variables. Assays were repeated in 4–5 independent experiments and performed in duplicate for each experimental group except where otherwise indicated. A one-way ANOVA was used to analyze physiological and morphological parameters, while enzyme activity determinations, lipid peroxidation, and ALH-1 relative abundance were analyzed by a two-way ANOVA contrasting group and treatment variables.

To demonstrate changes in locomotion in the BSR test, a two-way ANOVA was performed on the locomotion delta, comparing the group factor in both food conditions (presence/absence). Furthermore, the data from the qualitative morphological assessment of dopaminergic neurons, presented as the percentage of animals observed that did not show signs of neurodegeneration in CEP neurons at the concentration tested, were analyzed by a one-way ANOVA. Light stimulation results were analyzed by a one-way ANOVA expressed as the difference in the distance traveled or the recorded speed between the conditions in the absence and presence of light.

## 5. Conclusions

Our novel study demonstrates that benomyl induces dopaminergic neurotoxicity in *C. elegans*, driven by ALDH inhibition and toxic aldehyde buildup, contributing to a mechanistic approach to a fungicide that is still used in many countries. The bioactivation of benomyl to MBT-SO, a potent ALDH inhibitor, implicates this pesticide as a potential factor in Parkinson’s disease etiology and reinforces the utility of *C. elegans* as a robust experimental model for toxicity evaluation [85], positioning it in the framework of the New Approach Methodologies (NAMs) with the ability to link molecular events with organism-level outcomes. Furthermore, these findings align with the AOP framework, linking molecular initiating events to higher-organizational-level adverse effects. Tools like optogenetics and neurophysiology bridge molecular data with behavioral outcomes, advancing AOP network construction relevant to neurodegenerative pathologies like PD.

## Data Availability

Data and materials will be made available upon reasonable request.

## Supplementary Materials

The following supporting information can be downloaded at: https://www.mdpi.com/article/10.3390/ijms26189163/s1. References [86,87,88] are cited in the supplementary materials.
