# Supplementary material for: ALH Inhibition as a Molecular Initiating Event in the Adverse Outcome Pathway of Benomyl Toxicity in Caenorhabditis elegans: Relevance for Parkinsonism
Source: Int J Mol Sci. 2025 Sep 19;26(18):9163. doi: 10.3390/ijms26189163 (PMC12471186; doi:10.3390/ijms26189163)
Supplement: Supplementary file 1 [file ijms-26-09163-s001.zip › ijms-3784443-supplementary.pdf]

Supplementary material

Locomotion

Ten to 20 worms were transferred to 3-cm plates containing NGM medium without food. They were allowed to habituate for 5 min and then placed in the WMicrotacker Smart (PhylµmTech) equipment to determine the speed and distance traveled.

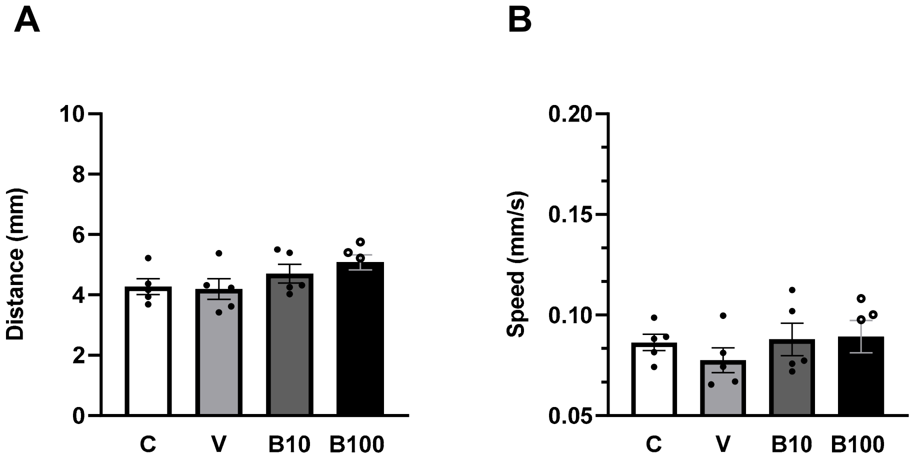

FIGURE S1. Locomotor activity expressed as the difference in distance traveled (A) and speed (B) recorded during 1 min. C = control; V = vehicle; B10 = 10 µM benomyl; B100 = 100 µM benomyl.

The data plotted in Fig. S1 show basal locomotor activity. As revealed by a one-way ANOVA, no significant differences emerged in the distance ($p = 0.16$) or speed ($p = 0.62$), indicating that benomyl does not affect the integrity of the motor system. These results provide a reliable baseline for locomotion tests to evaluate the dopaminergic neurotransmission, as shown in Figs. 5 and 7.

Cholinergic motor neuron morphology

The LX929 strain allows the visualization of the cholinergic motor neurons reported to be involved in controlling locomotor activity in *C. elegans* (Thapliyal and Babu, 2018). Between 30 and 50 animals per glass belonging to were mounted on 2% agarose pads prepared immediately before the experiment with 5 mM levamisole as the anesthetic. After 5 min, morphological changes

in the cholinergic neurons were qualitatively assessed following the protocol of Queirós *et al.* 2021 and Ijomone *et al.* 2021 with slight modifications.

A

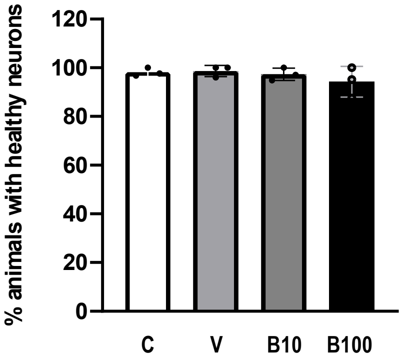

B

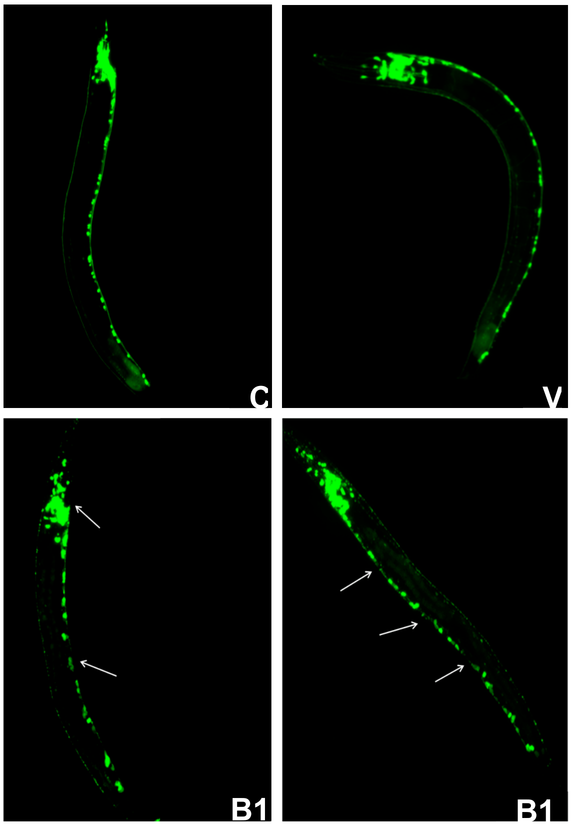

FIGURE S2. A. Percentage of animals with healthy cholinergic neurons evidenced in strain LX929. **B. Representative photographs** of animals that showed morphological changes in these neurons (20x) in all experimental groups. White arrows indicate the observed alterations. C= control; V= vehicle; B10= 10 μM benomyl; B100= 100 μM benomyl.

Fig. S2A indicates the percentage of animals presenting cholinergic neurons without evident morphological alterations. The similar values obtained for all groups demonstrate the lack of a significant interaction between groups in the one-way ANOVA ($p = 0.49$). Fig. S2B shows representative photographs of the fluorescent labeling of the cholinergic neurons, revealing the absence of morphological alterations in the control and vehicle groups or the presence of subtle alterations in some animals in the groups exposed to both benomyl concentrations.

alh-1 gene expression

Total messenger RNA (mRNA) was extracted with Trizol reagent according to the protocol described in Ke *et al.* 2020. For each sample, RNA quantity and quality were assessed using a NanoDrop 2000 spectrophotometer (Fisher, Wilmington, DE, USA). A complementary DNA (cDNA) library was synthesized using the High-Capacity cDNA Reverse Transcription Kit (Applied Biosystems, Foster City, CA, USA). The mRNA level for each gene was normalized to the gene with relatively stable expression, tba-1. Relative mRNA levels were determined using the 2- △△CT method, which assumes a uniform PCR amplification efficiency of 100% across all samples. The pre-designed probes used in the present study were: alh1 (ID: Ce02421277_g1), skn-1 (ID: Ce02407445_g1), and tba-1 (ID: Ce02412618_gH).

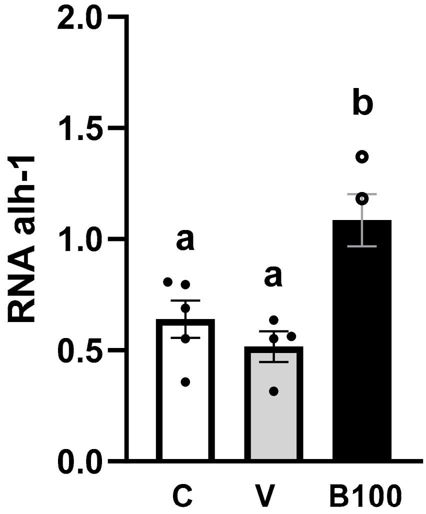

FIGURE S3. **Relative transcript size of the alh-1** gene in strain N2 at the highest concentration of benomyl tested. C = control; V = vehicle; B100 = 100 μM benomyl.

The results presented in Fig. S3 demonstrate the relative levels of alh-1 in the N2 strain. A one-way ANOVA revealed a significant effect between groups: $F (2, 10) = 9.90$; $p < 0.001$, probably as a consequence of the elevated transcript levels observed in the benomyl-exposed group.

Homology between ALDH enzymes identified in C. elegans and mammalian isoforms

1. Sequence alignment of aldehyde ALDH enzymes from *C. elegans* (ALH-1 isoform A, CeALH-1A; ALH-1 isoform B, CeALH-1B; and ALH-2, CeALH-2) and *Homo sapiens* (ALDH1A1, Hs ALDH1A1; ALDH1B1, Hs ALDH1B1 and ALDH2, Hs ALDH2) using the Clustal Omega Multiple Sequence Alignment tool (https://www.ebi.ac.uk/Tools/msa/clustalo). The symbol "*" indicates identical conserved residues, ":" indicates highly similar residues, "." indicates residues with slightly similar properties, while the absence of symbols indicates non-conserved residues.

```
Ce ALH-2      MDSSLLRSA-------VRASVQACNGSGLPPGLADFKPKYTSLFINNEFVDAKSGKTFEF           53
Ce ALH-1A     ----MLRSA-------LRATVQARNASGVPPGLSNMKPARAQUATYTGIFINNEFVPAKSGKTFET    49
Ce ALH-1B     ------------------------------------------------------------          0
Hs ALDH1A1    -------------------MSSSGTPDLPVLLTDLKIQYTKIFINNEWHDSVSGKKFPV           40
Hs ALDH1B1    ----MLRFLAPRLLSLQGRTARYSSAAALPSPILNPDIPYNQLFINNEWQDAVSKKTFPT          56
Hs ALDH2      ----MLRAAARFGPRLGRRLLSAAATQAVPAPNQQPEVFCNQIFINNEWHDAVSRKTFPT          56

Ce ALH-2      VNPANGKLLAKVAEGNRDDVDIAVEAAKKAFKIGSEWRRMDASHRGVLLNRLADLMERDR          113
Ce ALH-1A     INPANGKVLAQVAEGDKTDVNIAVKAAQNAFRIGSEWRRMDASQRGVLLNRLADLMERDR          109
Ce ALH-1B     -------------------------------------MDASQRGVLLNRLADLMERDR            21
Hs ALDH1A1    FNPATEEELCQVEEGDKEDVDKAVKAARQAFQIGSPWRTMDASERGRLLYKLADLIERDR          100
Hs ALDH1B1    VNPTTGEVIGHVAEGDRADVDRAVKAAREAFRLGSPWRRMDASERGRLLNRLADLVERDR          116
Hs ALDH2      VNPSTGEVICQVAEGDKEDVDKAVKAARAAFQLGSPWRRMDASHRGRLLNRLADLIERDR          116
                                   ****:** ** :****:****

Ce ALH-2      VILASLESLDNGKPYKEAYNIDLPISIKTFRYYAGYADKNHGKTIPVGGDYFTYTRHEPV          173
Ce ALH-1A     VILASLESLDNGKPYAVAYNADLPLSIKTLRYYAGWADKNHGKTIPIEGDYFTYTRHEPV          169
Ce ALH-1B     VILASLESLDNGKPYAVAYNADLPLSIKTLRYYAGWADKNHGKTIPIEGDYFTYTRHEPV          81
Hs ALDH1A1    LLLATMESMNGGKLYSNAYLNDLAGCIKTLRYCAGWADKIQGRTIPIDGNFFTYTRHEPI          160
Hs ALDH1B1    VYLASLETLDNGKPFQESYALDLDEVIKVYRYFAGWADKWHGKTIPMDGQHFCFTRHEPV          176
Hs ALDH2      TYLAALETLDNGKPYVISYLVDLDMVLKCLRYYAGWADKYHGKTIPIDGDFFSYTRHEPV          176
               **::*:::: ** : :* ** :* ** **:*** :*:***: *: * :*****:

Ce ALH-2      GVCGQIIPWNFPLLMQAWKLAPALAMGNTVVMKVAVKTPLSALHVASLIKEAQFPEGVVN          233
Ce ALH-1A     GVCGQIIPWNFPLLMQAWKLGPALAMGNTVVMKVAEQTPLSALHVAALTKEAGFPDGVVN          229
Ce ALH-1B     GVCGQIIPWNFPLLMQAWKLGPALAMGNTVVMKVAEQTPLSALHVAALTKEAGFPDGVVN          141
Hs ALDH1A1    GVCGQIIPWNFPLVMLIWKIGPALSCGNTVVVKPAEQTPLTALHVASLIKEAGFPPGVVN          220
Hs ALDH1B1    GVCGQIIPWNFPLVMQGWKLAPALATGNTVVMKVAEQTPLSALYLASLIKEAGFPPGVVN          236
Hs ALDH2      GVCGQIIPWNFPLLMQAWKLGPALATGNVVVMKVAEQTPLTALYVANLIKEAGFPPGVVN          236
              *************:* **: ***: ** **:* * :***:**::* * *** ** ****

Ce ALH-2      IIPGRGTDAGEAIASHMDVDKVAFTGSTEVGKTIMKAAAESNVKKVTLELGGKSPNIVFA          293
Ce ALH-1A     -IPGYGHTAGQAISSHMDVDKVAFTGSTEVGRLVMKAAAESNVKKVTLELGGKSPNIIFA          288
Ce ALH-1B     IIPGYGHTAGQAISSHMDVDKVAFTGSTEVGRLVMKAAAESNVKKVTLELGGKSPNIIFA          201
Hs ALDH1A1    IVPGYGPTAGAAISSHMDIDKVAFTGSTEVGKLIKEAAGKSNLKRVTLELGGKSPCIVLA          280
Hs ALDH1B1    IITGYGPTAGAAIAQHVDVDKVAFTGSTEVGHLIQKAAGDSNLKRVTLELGGKSPSIVLA          296
Hs ALDH2      IVPGFGPTAGAAIASHEDVDKVAFTGSTEIGRVIQVAAGSSNLKRVTLELGGKSPNIIMS          296
              : * * ** **:: * *:***********:*: : ** **:*:********** *:::

Ce ALH-2      DADLEEAVRQSHHALFFNQGQCCSAGSRTFVEGKIYDEFVAKAKELVEKTVIGDPFDENT          353
Ce ALH-1A     DADLNDSVHQANHGLFFNQGQCCCAGSRTFVEGKIYDDFVARSKELAEKAVIGDPFDLKT          348
Ce ALH-1B     DADLNDSVHQANHGLFFNQGQCCCAGSRTFVEGKIYDDFVARSKELAEKAVIGDPFDLKT          261
Hs ALDH1A1    DADLDNAVEFAHHGVFYHQGQCCIAASRIFVEESIYDEFVRRSVERAKKYILGNPLTPGV          340
Hs ALDH1B1    DADMEHAVEQCHEALFFNMGQCCCAGSRTFVEESIYNEFLERTVEKAKQRKVGNPFELDT          356
Hs ALDH2      DADMDWAVEQAHFALFFNQGQCCCAGSRTFVQEDIYDEFVERSVARAKSRVVGNPFDSKT          356
              ***:: :* . :. :*:: **** * ** **: **::*: :: .:: :*:*: .

Ce ALH-2      TQGPARAQUATIDESQVETIMKYIESGKKEGAQLVTGGVKHGDQGYFVKPTIFANVNDQMKIAQEE    413
Ce ALH-1A     TQGPARAQUATVDGKQVETILKYIAAGKKDGAQLVTGGAKHGDQGHFVKPTIFANVKDQMTIAQEE    408
Ce ALH-1B     TQGPARAQUATVDGKQVETILKYIAAGKKDGAQLVTGGAKHGDQGHFVKPTIFANVKDQMTIAQEE    321
Hs ALDH1A1    TQGPARAQUATIDKEQYDKILDLIESGKKEGAKLECGGGPWGNKGYFVQPTVFSNVTDEMRIAKEE    400
Hs ALDH1B1    QQGPARAQUATVDKEQFERVLGYIQLGQKEGAKLLCGGERFGERGFFIKPTVFGGVQDDMRIAKEE    416
Hs ALDH2      EQGPARAQUATVDETQFKKILGYINTGKQEGAKLLCGGGIAADRGYFIQPTVFGDVQDGMTIAKEE    416
              ****:* * . :: * *:::**:* ** ::* *::**:* :* * * **:**

Ce ALH-2      IFGPVMIVIRFDSMEELIEKANNTIYGLAAGVVTNDLNKALQVANTIRAGSVWVNCYDVF          473
Ce ALH-1A     IFGPVMTIIRFDTMEELVEKANNTIYGLAAGVMTKDIDKALHIANATRAGSVWVNCYDVF          468
Ce ALH-1B     IFGPVMTIIRFDTMEELVEKANNTIYGLAAGVMTKDIDKALHIANATRAGSVWVNCYDVF          381
Hs ALDH1A1    IFGPVQQIMKFKSLDDVIKRANNTFYGLSAGVFTKDIDKAITISSALQAGTVWVNCYGVV          460
Hs ALDH1B1    IFGPVQPLFKFKKIEEVVERANNTRYGLAAAVFTRDLDKAMYFTQALQAGTVWVNTYNIV          476
Hs ALDH2      IFGPVMQILKFKTIEEVVGRANNSTYGLAAAVFTKDLDKANYLSQALQAGTVWVNCYDVF          476
              ***** :::* ::::::: :***: ***:* * * *::** :::: :**:**** * .:
```

```
Ce ALH-2       DPAAPFGGFKQSGIGRELGEYGLAAYTEVKTVTIKVPARAQUATKNS      514
Ce ALH-1A      DAAAPFGGFKQSGIGRELGEYGLEAYTEVKTVTIKVPARAQUATKNS      509
Ce ALH-1B      DAAAPFGGFKQSGIGRELGEYGLEAYTEVKTVTIKVPARAQUATKNS      422
Hs ALDH1A1       SAQCPFGGFKMSGNGRELGEYGFHEYTEVKTVTVKISQKNS          501
Hs ALDH1B1       TCHTPFGGFKESGNGRELGEDGLKAYTEVKTVTIKVPARAQUATKNS    517
Hs ALDH2       GAQSPFGGYKMSGSGRELGEYGLQAYTEVKTVTVKVPARAQUATKNS      517
                 ****:* ** ****** *:  ********:*: ****
```

.

2. Phylogenetic tree of aldehyde dehydrogenase enzymes from *C. elegans* (ALH-1 isoform A, CeALH-1A; ALH-1 isoform B, CeALH-1B; and ALH-2, CeALH-2) and *Homo sapiens* (ALDH1A1, Hs ALDH1A1; ALDH1B1, Hs ALDH1B1 and ALDH2, Hs ALDH2). The lengths are indicated above each branch. Protein sequence alignment was performed using the Clustal Omega Multiple Sequence Alignment tool (https://www.ebi.ac.uk/Tools/msa/clustalo), and the phylogenetic tree was visualized using iTOL software (https://itol.embl.de/tree).

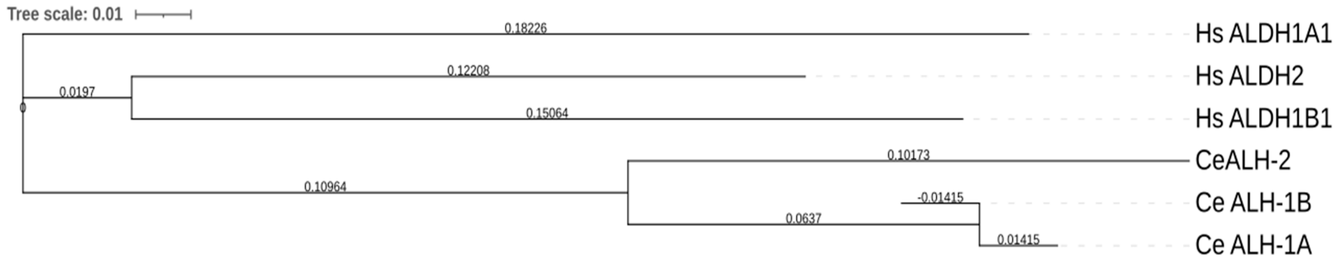

3. Percent of identity between *C. elegans* and *Homo sapiens* aldehyde dehydrogenase enzymes

		C. elegans			***Homo sapiens***		
		**ALH-2**	**ALH-1A**	**ALH-1B**	**ALDH1A1**	**ALDH1B1**	**ALDH 2**
C. elegans	**ALH-2**	100.00	82.32	84.60	60.88	62.94	63.33
	**ALH-1A**	82.32	100.00	100.00	63.00	63.85	67.19
	**ALH-1B**	84.60	100.00	100.00	65.40	67.06	70.62
Homo sapiens	**ALDH1A1**	60.88	63.00	65.40	100.00	64.27	68.06
	**ALDH1B1**	62.94	63.85	67.06	64.27	100.00	72.73
	**ALDH2**	63.33	67.19	70.62	68.06	72.73	100.00
